# Compensatory behavior of physical activity in adolescents – a qualitative analysis of the underlying mechanisms and influencing factors

**DOI:** 10.1186/s12889-023-17519-1

**Published:** 2024-01-11

**Authors:** Franziska Beck, Brittany Amel Swelam, Ulrich Dettweiler, Claus Krieger, Anne Kerstin Reimers

**Affiliations:** 1 Department of Sport Science and Sport, Friedrich-Alexander-Universität Erlangen-Nürnberg, Erlangen, Bavaria, 91058 Germany; 2 Institute for Physical Activity and Nutrition, Deakin University, Burwood, 3125 Australia; 3 Cognitive and Behavioral Neuroscience Lab, University of Stavanger, 4036 Stavanger, Norway; 4 Department of Languages and Aesthetic Disciplines Education, Universität Hamburg, Hamburg, 20148 Germany

**Keywords:** Physical activity, Compensation, Youths, Qualitative

## Abstract

**Introduction:**

Compensatory behavior of physical activity (PA) based on the ActivityStat hypothesis in adolescents is scarcely investigated, and existing studies showed inconclusive results. Understanding the compensatory behavior in a holistic way is important as this can help to improve intervention outcomes and thus, increase the PA levels in adolescents. Thus, the aim of the present study is to investigate the occurrence, direction, timeframe, and ratio of habitual activity compensation in adolescents. Furthermore, we want to identify the awareness of compensation and factors that influence compensatory behavior.

**Methods:**

The present qualitative study used a mixed methods crossover analysis design. Participants (*N* = 15, 8 boys and 7 girls) were adolescents aged 11–15 years (mean age 13.04 ± 1.28). They provided a habitual weekly schedule with habitual/regular activities and their intensity. Participants then kept an activity diary over one week to capture their actual behavior. After that, data were compared and deviations > ±20% were considered as compensation opportunities. On this basis, deviations were descriptively analyzed for compensatory behavior and were coded as positive and negative compensatory behavior. Further, for each compensation, the ratio of compensation (*MET-minutes of the compensating activity/MET-minutes of the activity that was compensated*) was calculated. Additionally, interviews were conducted to explore perceptions and influencing factors for (no) compensation.

**Results:**

Overall, 198 compensation opportunities were identified with deviations greater ± 20%. Of these, 109 opportunities were compensated overall (69 within-day, 40 between-day). Negative compensation took place in 57 opportunities and 52 opportunities were compensated positively. Most of the deviations were overcompensated (compensation/deviation > 100%). About half of the adolescents (*N* = 8) were not aware about their compensatory behavior, and only one boy was aware of all his compensatory behavior. The most mentioned influence for positive compensation were social support by friends and good weather. As influencing factors for negative compensation, tiredness as well as no need for movement were mentioned predominantly. No negative compensation occurred because adolescents wanted to stick to their routines or participated in hedonistic activities.

**Discussion:**

Summarizing the findings, the present study delivered new insights into the field of compensatory behavior in adolescents. Nevertheless, compensatory behavior was not consistently observed regarding the occurrence of compensation, direction, timeframe and ratio. However, social support appears to be an important factor to compensate positively or to avoid a negative compensatory behavior. Further, it seems to be helpful to support individuals in their search for hedonistic activities as well as in the establishment of routines.

**Supplementary Information:**

The online version contains supplementary material available at 10.1186/s12889-023-17519-1.

## Introduction

Physical activity (PA) is associated with several health benefits in children and adolescents [1–5] like improved physical fitness [6–8], a reduction in the risk of obesity, and improvements in cardiovascular and cardiometabolic health [1, 4, 9]. However, children and adolescents in many regions worldwide do not meet the WHO recommendations for PA [10–13]. In addition, with increasing age, the prevalence of compliance to the WHO PA guidelines decreases [14–16]. Despite numerous efforts to promote PA among children across different settings, including schools, the family environment or local community settings, the effectiveness in the short and long term has widely varied and is not sufficient [17, 18].

To successfully harness the health effects of PA (within interventions), it is important to fully understand the determinants. In this regard, some studies have focused on potential biological influences of PA, specifically the potential effects of intrinsic biological control on regular activity [19, 20]. This issue was highlighted with Rowland's seminal ActivityStat hypothesis paper [20], which has since gained prominence in the literature. This hypothesis focuses on the potential effect of intrinsic biological control that may underpin PA and energy expenditure (EE). In particular, the ActivityStat hypothesis posits that an imposed increase or decrease in PA in one domain/timespan might induce a compensatory change in the opposite direction in another domain/timespan in order to maintain a stable level of PA or EE over the time [21]. However, until now, reviews on compensatory behavior studies have found inconsistent results regarding the support for the ActivityStat hypothesis [21–23].

Previous research has suggested that perceptions of PA can be an important indicator of activity behavior [24, 25]. Therefore, understanding adolescents’ perceptions of their activity behavior may be important in understanding the causes of their compensatory behavior. Specifically, this may provide further insights into the malleability of the ActivityStat hypothesis [26–28], as well as an understanding of whether external factors (e.g., environmental, interpersonal, etc.) may influence compensatory responses. Until now, few studies have examined perceptions of compensation and/or mechanisms of compensation (i.e., whether adolescents perceive that they compensate their activity and if so, what factors influence this) [29, 30]. Furthermore, relatively little research has been done on adolescents [29]. However, this was not a purpose-designed study to explore the perceptions of compensation in this age group. A previous study examining perceptions and mechanisms of compensation in primary school children identified several influences on compensation, as well as an awareness of within-day PA compensation [31]. Whilst this study identified effects of environmental (e.g., home structure), interpersonal (e.g., co-participation), psychological, and physiological factors, it does not focus on the PA amount of deviation from the habitual PA that leads to a compensatory behavior as well as the amount of compensatory PA. In addition, this study occurred in pre-adolescent participants.

Therefore, the aim of the present study was to investigate whether adolescents compensate for increases or decreases in their habitual PA within-day/between-days and whether they perceive this directly. Furthermore, we wanted to identify the mechanisms and factors that influence compensatory behavior.

## Methods

### Study design

The present qualitative study used a mixed methods crossover analysis design [32]. The study was approved by the local Ethics Committee of the Friedrich- Alexander-Universität Erlangen-Nürnberg (Ref. No. 23–31-S) and was in accordance with the 1964 Declaration of Helsinki. All participants and their legal guardians provided written informed consent for study participation.

### Participants

Adolescents aged 11–15 years and living in Germany, were recruited via personal contacts, various youth institutions as well as sports clubs and other leisure time instances in the region of Erlangen (Bavaria). These participants were recruited using theoretical sampling methods [33], and were selected in accordance with our proposed analysis processes and theoretical underpinnings. This included ensuring the samples contain diversity with respect to socioeconomic status, migration background, sex/gender and environmental conditions (e.g., urban and rural living locations). In more detail, we tried to find an equal amount of boys and girls for each categorie (sex/gender, age, migration background, living area, and school type). Our final sample consisted of 15 adolescents. Adolecents could not be part of the study if they had a physical disability or if they didn’t have a smartphone. All participants received 10 Euro as an incentive. 

### Data collection

First of all, sociodemographic data, like age, gender, educational level, and residential area, was collected from both adolescents and their parents via a short questionnaire sent per e-mail. Data collection took place in spring 2023 and had three parts. First, participants were asked to fill a schedule with all of their habitual/typical physical activities in a given week via a plain document. Within the information letter and the example of a weekly schedule (Additonal file 1) participants received additional information for the weekly schedule. Examples of activities were provided to increase clarity. Nevertheless, if adolescents needed help to fill out the habitual schedule, they could reach out to our research team or ask their parents. The following week, participants were asked to track their activities via a smartphone-based diary app (ClueTec GmbH; https://www.cluetec.de). To use the diary, adolescents received a QR-code that made an installation of the programmed diary available. To minimize recall bias, adolescents were instructed to track their activities ideally immediately after the activity, however, if this was not possible, then always as soon as possible. Following the completion of the weekly schedule and the movement diary by participants, the research team assessed the schedule and diary for potential compensatory behavior. Then, participants engaged in an online interview via Jitsy-Meet (8 × 8 Inc.). Interviews were anticipated to take around 30 min to complete, however, a 60 min appointment was scheduled to allow for potential deviations. After giving informed consent and agreeing on an appointment, each participant received an individual link for an online meeting to conduct the interview. Participants were able to complete their interview from any desired place so long as they had a stable internet connection and quiet surrounding. Before the start of the recording, the objective and the interview procedure were explained and participants were reassured of the voluntary nature of their involvement and their right to refuse to answer any questions. After clarifying any questions that participants had, the audio recording device was turned on and the interview began. All participant data was de-identified to ensure anonmyity and participant names were not recorded during interviews. 

### Measures

#### Weekly activity schedule (habitual PA)

Participants provided information about their habitual (active and non active) activties within one week. They gave information about the time of the activity, the duration, type and intensity. To ensure participant clarity and understanding, participants received a template containing two examples (Additional file 1).

#### Smartphone-based activity diary (actual PA)

Participants were asked to track their activities with a smartphone based diary app (ClueTec GmbH). Participants were asked to track each PA they did, and upon recording the activity in the diary, were asked the following questions: What did you do exactly? For how long? How was the intensity (LPA, MPA, VPA)?

#### Semi-structured interview – interview guideline

The interview guideline was based on the comparison of the weekly activity schedule and the diary (Additional file 2). This allowed for the identification of compensatory behavior. The specific questions that were asked were dependent on whether compensation had occurred or not, with subsequent questions relating to perceptions of compensation, and influencing factors for the compensatory behavior (or not) (e.g., why did you do more/less at time point X and why subsequent less/more than usual at timpoint Y? Did you perceive that you compensate your activity?). 

### Data analysis

#### Descriptive analysis of deviation and compensation with weekly activity schedule (habitial PA) and activity diary (actual PA)

To explore compensation, MET-minutes were caluclated for each habitual physical activities (derived from the weekly schedule) and actual physical activities (derived from the smartphone diary). Sedentary behavior was not considered in our data analysis that focus on activity compensation. The Youth Compendium of PA [34] was utilised to calculate the MET cost for each activity (if an activity wasn’t listed in the Youth Compendium, the MET cost was estimated based on similar activities), then subsequently multiplied by the duration of the activity to determine the MET-minutes for each activity. To determine wether deviation had occurred, the difference between the actual MET-minutes of the diary activity and the habitual MET-minutes of the weekly schedule’ activity at the same time period (MET-minutes_actual – MET-minutes_habitual) as well as the percentage of this difference were calculated. 

To define opportunities where compensatory behavior could occur, the intra-individual variability was considered. Therefore, to be identified as a possible compensatory opportunity, the difference between habitual MET-minutes in the weekly schedule’s activity and actual MET-minutes from the diary’s activity at the same time point had to be at least ± 20% different from the habitual MET-minutes [26, 35] (Additional file 3). Possible compensatory opportunities were then assessed to determine if compensation had occurred (or not) within that same day (within-day) and/or the next day (between-day), and in which ‘direction’ (i.e., positive or negative compensation). The compensatory definitions have been provided in Table 1.

**Table 1:** Definition of compensation related terms

Term	Defintion
Deviation	The difference between the actual MET-minutes of the diary and the habitual MET-minutes of the weekly schedule (MET-minutes_actual – MET-minutes_habitual)
Compensation opportunitiy	Participants had less/more Metabolic equivalent (MET)-minutes activity in the 'actual' week than their 'habitual' week, this was classified as a 'compensation opportunity'
Positive compensation	In the case that participants completed additional non-habitual MET-minutes in the actual week following this change, this was considered 'positive compensation'
Negative compensation	In the case that participants completed less non-habitual MET-minutes in the actual week following this change, this was considered 'negative compensation'
Ratio of compensation	The ration of compensation is calculated by following equation: *MET-minutes of the compensating activity /MET-minutes of the activity that was compensated* Ratio of compensation is distinguished between partial compensation (low: 0–49.9%. medium: 50–74.9% and high: 75–99.9%) and overcompensation (≥ 100%)

After identification of compensation in the participants, the ratio of compensation (MET-minutes of the compensating activity /MET-minutes of the activity that was compensated) was calculated and we distinguished between partial compensation (low: 0–49.9%. medium: 50–74.9% and high: 75–99.9%) and overcompensation (≥ 100%) [35]. 

**Fig. 1:**
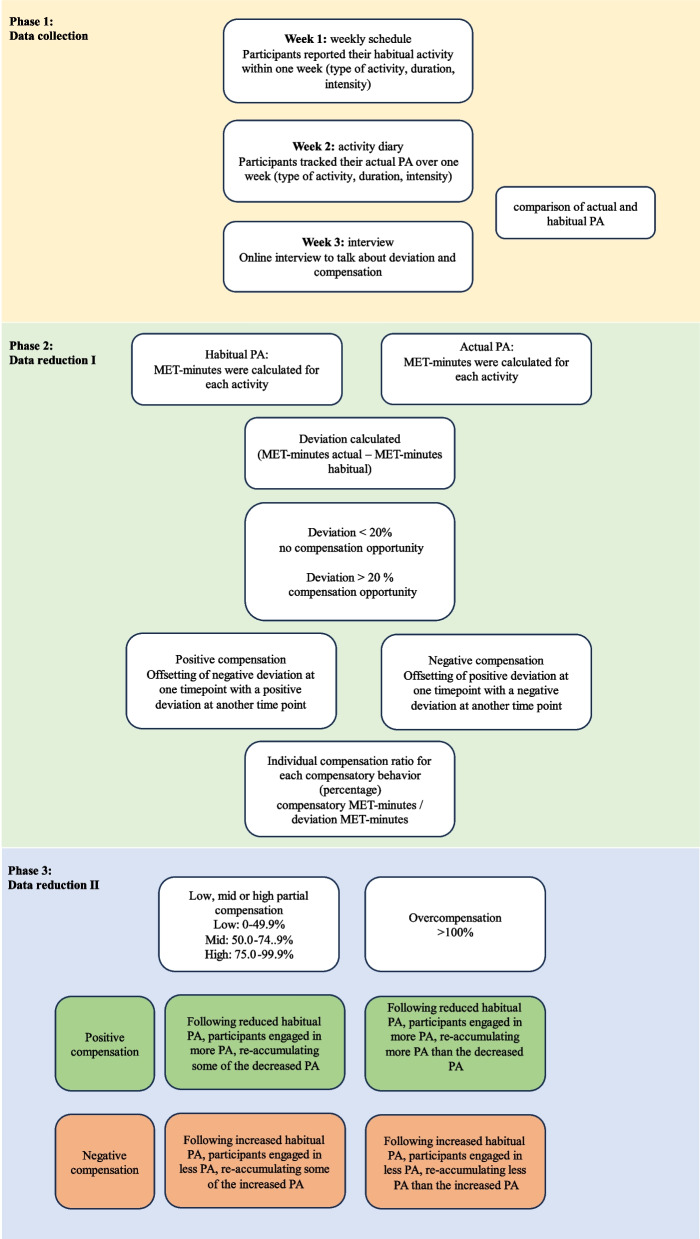
Data collection and data reduction process

On overview of data collection and data reduction process can be seen in Fig. 1.

#### Qualitative data – interviews

Interviews were transcribed verbatim according to DresingPehl [36] using f4transcript (audiotranskription.de). The resulting text files were checked for correctness. For the purpose of this paper, we translated the example quotes from German to English. 

Using the transcribed interview text, qualitative content analysis [37–39] was performed in QCAmap (https://www.qcamap.org/ui/en/home) [40]. Due to the category-guided approach, the analyses of the interviews was limited to verbal statements, whilstpara-verbal (e.g., body language, tone, etc.) information was not assessed, as it was deemed irrelevant for the extraction of information with regard to the research question [41]. Initial coding of interviews was based on principles derived from the ActivityStat hypothesis [21, 28] and considerations from previous qualitative studies examining awareness and potential mechanisms of compensation in elementary school children [31] and adults [30]. This analyzing stategy focused on awareness of compensatory behavior, influences for compensation as well as for not compensating. One author (FB) and a trained research assistant student conducted deductive analyses of the transcripts for awareness of compensation (“yes” or “no”) and inductive for influencing factors for (no) compensation. The codes were developed and categorized and further discussed with the research team.

## Results

### Sample characteristics

The mean age of the 15 adolescents (8 boys and 7 girls) was 13.04 (*SD* = 1.28). For the age of 15, we only had one boy and no girl. Overall, about half (*N* = 6; 3 boys and 3 girls) of the participants attended a secondary school (Realschule), whilst the majority (*N* = 8; 4 boys and 4 girls) of remaining participants attended high school (Gymnasium). For middle school, we only included one boy. An imbalance can be seen in the type of residence for rural areas (5 boys and only 1 girl live in rural areas). The majority of the adolescents’ parents were born in Germany, with the exception of two participants with either one (*N* = 1) or both (*N* = 1) parents born outside of Germany. Overall, the adolescents accumulated in the diary 661.0 (293.6) MET-minutes per day in the diary, indicating an active sample (moderate PA = 3–6 MET-minutes, vigorous PA > 6 MET-minutes) [42]. Further information can be seen in Table 2.

**Table 2:** Sample description

	**Overall**	**Boys**	**Girls**
**Adolescents**			
N (%)	15 (100)	8 (53)	7 (47)
boys			
girls			
Age (mean SD)	13.04 (1.28)	12.99 (1.46)	13.10 (1.17)
11 (N, %)	3	2 (66.7)	1 (33.3)
12	4	2 (50.0)	2 (50.0)
13	5	2 (40.0)	3 (60.0)
14	2	1 (50.0)	1 (50.0)
15	1	1 (100)	
*School type*			
Middle School (Haupt/Mittelschule)	1 (7)	1(12.5)	0
Secondary School (Realschule)	8 (53.3)	4 (50.0)	4 (57.1)
High School (Gymnasium)	6 (40)	3 (37.5)	3 (42.9)
*Urbanisation*			
City > 100.000 inhabitants	5 (33)	2 (25.0)	3 (42.8)
Medium sized town 20.000–100.000 inhabitants	4 (27)	1 (12.5)	3 (42.8)
Rural area/village	6 (40)	5 (62.5)	1 (14.4)
*Migration Background*			
None	13 (87)	6 (75.0)	7 (100)
One custodian	1 (7)	1 (12.5)	0
Both custodian	1 (7)	1 (12.5)	0
*Activity Level*			
Diary MET Min per day (mean; standard deviation)	661.0 (293.6)	796.4 (304.9)	506.3 (183.4)
Range (min – max)	227.3 – 1175.4	389.1 – 1175.4	227.3 – 724.1
**Custodians**			
*Educational Level of custodian 1*			
(qualified) middle school degree	4 (26.7)	2(25.0)	2 (28.6)
High school degree	11 (73.3)	6 (65.0)	5 (62.5)
*Educational Level of custodian 2*			
Finished school without degree	1 (7.1)	1 (12.5)	0
Middle school degree	6 (42.9)	3 (37.5)	3(50.0)
General high school degree	7 (50.0)	4 (50.0)	3 (50.0)
*Employment of custodian 1*			
Part-time	9 (60.0)	3 (37.5)	6 (85.8)
Full-time	6 (40.0)	5 (62.5)	1 (14.2)
*Employment of custodian 2*			
Unemployed	1 (6.7)	1 (12.5)	0
Retiree	1 (6.7)	1 ()	0
Part-time	1 (6.7)	1 (12.5)	0
Full-time	11 (79.9)	5 (62.5)	6 (100)

### Data quality

Filling out the habitual weekly schedule took the adolescents around 30 min. For each activity that was tracked in the activity diary, adolescents needed 2–5 min. The weekly schedule contained no time gaps. In the activity diary, only PA was tracked. If there was something missing in the diary, which was supposed to be included, participants were contacted individually and asked if this hadn’t taken place or if it just had been forgotten.

### Descriptive compensation analysis

Overall, 198 opportunities were identified where participants had a (positive/negative) ± 20% deviation from the habitual PA (condition/perquisition for compensation). Among these, 114 (57.6%) of possible compensation opportunities occurred in boys, whereas 84 (42.4%) occurred in girls. Compensation occurred n 55.1% (*N* = 102) of the possible compensation opportunities. Boys compensated in 75 out of 114 (64.0%) possible compensation opportunities whereas girls compensated in 34 out of 84 (40.5%) possible compensation opportunities. Positive compensation occurred in 52 of the 198 opportunities (26.3%; boys, *N* = 41; girls, *N* = 11), whereas negative compensation occurred in 57 opportunities (28.8%; boys, *N* = 34; girls, *N* = 23).

#### Positive compensation

**Fig. 2:**
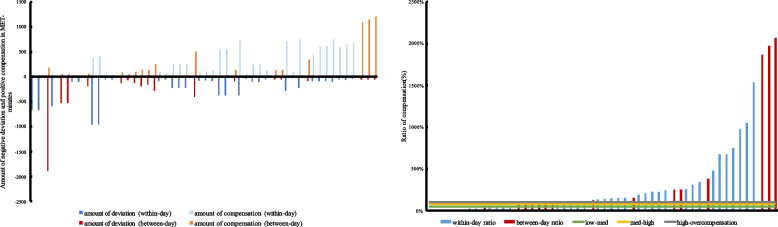
Overall amount of negative deviation and subsequent positive compensation (left) as well as ratio of compensation (right) with the thresholds for low, medium and high partial compensation as well as overcompensation. Blue colors indicate the within-day compensation and red colors the between-day compensations. Each bar represents one compensatory behavior

To compensate in a positive way, a negative deviation must have preceded, i.e., the actual PA at a given time point t is lower than the habitual PA at this time point. Details about the amount of MET-minutes for deviations and compensation overall, as well as for boys and girls can be seen in Additional file 4. Looking at the ratio of compensation, overall, it ranged between 6% (low partial compensation) and 2068% (overcompensation). In boys and girls, similar tendency can be seen (boys: 6% to 1533%, girls: 7% to 2068%). Figure 2 illustrates the amount of deviation and compensation for each occurred compensation (left) and the associated ratio of compensation (right). In addition, proportion of low, medium, high and overcompensation for negative compensation can be found in Additional file 5.

Lastly, regarding the timeframe, of all [*n* = 52] positive compensatory behaviors, 65.4% [*n* = 34] occurred within-day, whereas 34.6% occurred between-day (see Additional file 6). Between-day could either refer to successive days like Monday-Tuesday or occur over several day like Monday-Friday. Further, Fig. 2 shows the amounts of MET-minutes. Additional file 4 shows the amounts of MET-minutes for deviation and compensation within and between day. The compensation ratio ranged in boys within-day from 6 to 1533% and between-day the ratio of compensation was 11% to 377%. Ratio of compensation in girls ranged from 58 to 149% within-day and 7% to 2068% between-day.

#### Negative compensation

**Fig. 3:**
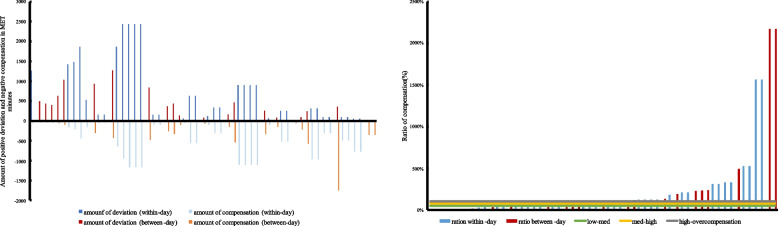
Overall amount of positive deviation and subsequent negative compensation (left) as well as ratio of compensation (right) with the thresholds for low, medium and high partial compensation as well as overcompensation. Blue colors indicate the within day compensation and red colors the between-day compensations. Each bar represents one compensatory behavior

In contrast, positive deviation leads to negative compensatory behavior. The amount of deviation and compensation can be seen in Additional file 4 as well as in Fig. 3. The ratio of compensation ranged between 4% (low partial compensation) and 2173% (overcompensation) (see Fig. 3). In boys, the ratio of compensation ranged from 7 to 525%, and in girls the range was from 4 to 189%. Furthermore, the proportions of low, medium, high and overcompensation for negative compensation can be found in Additional file 7.

Lastly, regarding the timeframe, of all [*n* = 57] negative compensatory behaviors, negative compensation occurred in 35 opportunities [61.4%] within-day, and in 38.6% between-day (see Additional file 6). Further, Fig. 3 shows the amounts of MET-minutes and Additional file 4 the amounts of MET-minutes for positive deviation and negative compensation within and between day. The ratio ranged in boys within-day from 14 to 525% and between-day the ratio of compensation was 7% to 490%. Ratio of compensation in girls ranged from 4 to 94% within-day and 8% to 189% between-day.

#### No Compensation

Deviations from the habitual PA level were not always compensated. Compensation [*n* = 198] did not occur in 89 (44.9%) of 198 possible opportunities. Of these 89 possible compensation opportunities where no compensation occurred, 85 (95.5%) were negative deviations whereas just 4 opportunities with a positive deviation were not compensated. In boys there were 39 opportunities that were not compensated (36 negative deviation, *N* = 36 [92.3%]; positive deviation, *N* = 3 [7.7%]) and in girls there 50 opportunities that were not compensated (49 negative deviation, *N* = 49 [98.0%]; 1 positive deviation, *N* = 1 [2.0%]). Details about the amount of deviation in MET-minutes can be found in Additional file 8.

### Qualitative Data

#### Interviews Topic 1: Awareness of compensation

The interviews were conducted on the comparison between habitual weekly schedule and activity diary. Regarding compensatory behavior, the participants were asked about the awareness of compensation. Amongst all compensatory behaviors, about half of the adolescents did not perceive that they had compensated (*N* = 8) (*“I wouldn’t call it a conscious decision to do sports, simply because I had time and she [her friend] also plays handball with me, she [her friend] also had nothing to do, so we just said, come on, let’s go outside for a bit and the weather was nice anyway”*). Compensation occurs more often spontaneously in the situation than it is consciously perceived even if the compensatory analysis indicated a compensation. However, less adolescents (*N* = 5) were aware of compensation in some opportunities, but not in all of their compensatory opportunities (example for awareness: Interviewer *“Okay so that was also a bit of a conscious decision that you said ‘wow, the day before was very exhausting, very active, now I’m going to slow down a bit’”* and Adolescent: *“Yes, yes, yes”;* example for no awareness of the same adolescent*:”It was a spontaneous decision. My friend wrote me on Saturday and asked if I would like to play football with her [friend] spontaneously. And since it was just nice weather, I went of course with the bike, so that’s how it came about from each other. And yes, but if she hadn’t asked me, I wouldn’t have gone anywhere either. I don’t think so.”*). Only one adolescent was aware of all the compensatory behavior he did (*“Yes, so it was quite consciously like ‘okay, before I sit in the car for eight, nine hours now, I try to move more than usual in the morning”*).

#### Interviews Topic 2: Influences for compensation

As the perception of compensation indicated, there were often situational influences for compensation. Overall, in the present study we distinguished between internal and external influencing factors for compensation. Further, the influences for compensation differed between positive and negative compensation (see Table 3).

**Table 3:**
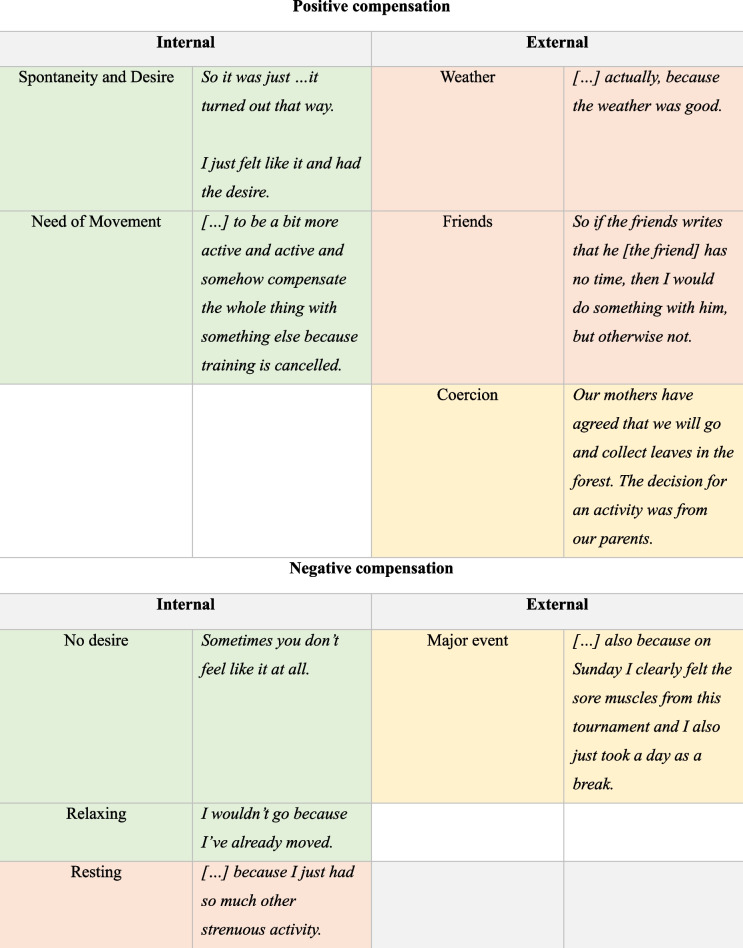
Influencing factors for compensation

Note: Red color = greatest importance, orange = medium importance and green = low importance

#### Positive Compensation—internal

Within the positive compensatory behavior, there were two internal and three external influencing factors identified each. As internal factors for positive compensatory behavior, the need of movement was one influencing factor mentioned by a few participants. This means that after a decrease of PA, participants felt a need for movement. This ended up in an extra activity or an increase of habitual PA (*“when I didn't have any training on Wednesdays, I had to do something for school that day, but I still went outside for a few minutes later, because I probably still had the urge to move, even though I already rode my bike today.”*). Next, adolescents mentioned being physically active after decreasing because of the desire to move in a spontaneous way. Adolescents said *“I just feel it in this moment to be active”* or *“it has turned out this way”* without given any conscious influencing factor.

#### Positive Compensation—external

Regarding external factors for a positive compensatory behavior, the most frequently mentioned influence was social support. Having a friend to be active with supported a positive compensation after a decrease in PA (*“My friend comes over to my house and asked me if I want to play football with her.”*). Good weather conditions were also cited as an influencing factor for compensation (*“in the evening I played outside because the weather was good”*). Adolescents perceived the weather as an incentive to be more active/do extra activity at another time after inactivity/less PA. The last external factor mentioned by the participants was the encouragement/enforcement of activity from the parents. One female participant mentioned that her mother/parents told her to go out because training was cancelled and thus organized a meeting with her friends and their parents to go in the forest and collect leaves (*“Our mothers actually made it out that we would then go and collect leaves. That was a decision made by the parents, that we should move around, go out, play and collect leaves.”).*

#### Negative Compensation – internal

Overall, three internal influences were identified for a negative compensation. First, participants mentioned that they were exhausted because of an increase of PA and thus, needed a rest (*“So I was very exhausted, especially in the legs, because I had been jogging two days before, then soccer tournament and then it was a bit exhausting, so I did less afterwards”*). Further, adolescents wanted to relax after additional PA because they felt like they had already done enough (*“I didn’t go [to training], because I've already moved”*) even if they didn’t feel really exhausted. In this context, a few adolescents also said that they didn’t have a desire to continue with the habitual activities after they had more or an extra activity and thus, they compensated negatively.

#### Negative Compensation – external

In addition, an external influencing factor for a negative compensation has also been identified. Participants took a rest from their habitual behavior and were more inactive after a big event like a tournament (*“So as far as a workout is concerned, also because on Sunday I clearly felt the sore muscles from this tournament and I also just took a day as a break, so to speak, after this day full of activities on Saturday”*).

#### Interviews Theme 3: Influences for not compensating

Besides influencing factors for compensating, our interviews identified a wide range of internal and external influences for not compensating, positive and negative. An overview can be seen in Table 4 and in the following paragraphs a more detailed explanation.

**Table 4:**
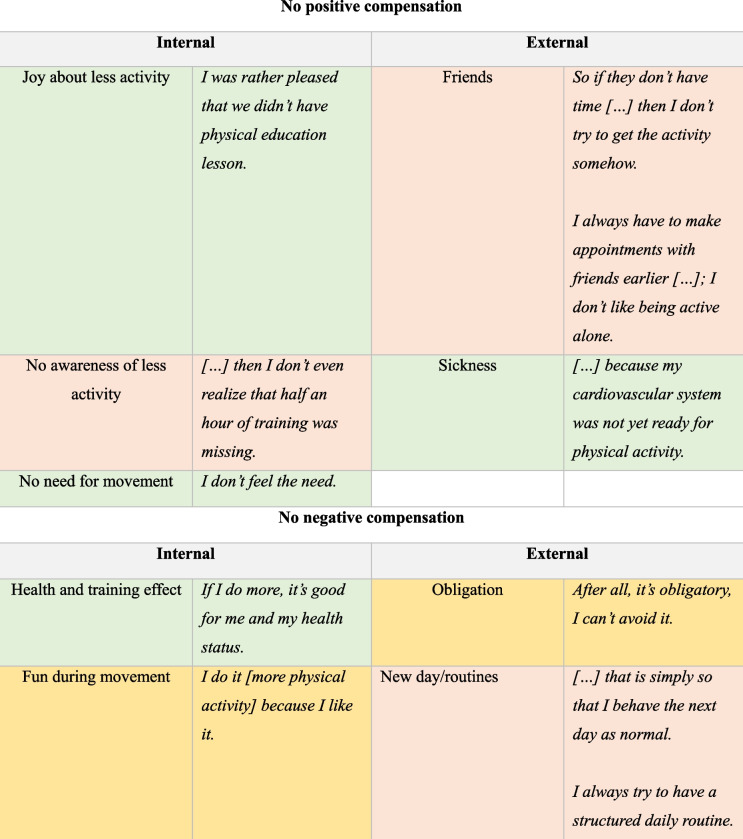
Influencing factors for no compensation

Note: Red color = greatest importance, orange = medium importance and green = low importance

#### Positive possible – internal

Besides the influences for compensation, the study also focused on influencing factors why adolescents did not compensate. The majority of adolescents didn’t see the need for more PA after they had less PA than usual (*“I don’t feel the need”*)*.* In some cases, they were happy about the spontaneous/unplanned reduction in habitual PA (*“it was not bad that training was cancelled”; “I was happy that physical education was cancelled and I then also no longer really had the need not to move”*) or were not aware of the lack of PA (*"then I don't even realise that half an hour of exercise is now missing."*).

#### Negative possible – internal

Opportunities with more PA than usual could have been compensated negatively, but there were also opportunities that were not compensated by less PA. Some adolescents mentioned that their lack of negative compensation was prompted by the fun/joy of movement related to the additional activity. In other words, they didn’t compensate for the activity because it was something they enjoy (*“I do it [jogging—even if there was more activity than usual in the morning] because I enjoy it.”*). Additionally, in some instances more PA than usual was perceived as profitable and good for health (*“I do more, because it’s good for my health”).*

#### Positive possible – external

One external influence why adolescents did not compensate the decreased activity is that they perceived a lack of social support. Thus, they decided to be rather inactive than being active alone (“*I always have to make appointments with friends earlier […] I don't like being active alone (so I did no activity)"; “So if they don’t have time […] then I don’t try to get the (decreased) activity somehow”*). Further, sickness could be a barrier to be active and thus, a loss of activity wasn’t compensated.

#### Negative possible – external

Some adolescents reported that they did not compensate negatively between-days because they thought of each day as a reset from the previous day (*“that is then simply so that I do it again the next day as normal.”; “No (I did not do less after an increase of PA), that's just the way it is then, that I just made more”.*) and thus complied with the typical structure of the following day (*“I always try to have a structure routine”*). Lastly, some reported that they did not compensate when activities were obligatory, such as taking part in physical education lesson (“*"After all, it's obligatory, I can’t avoid it."*).

## Discussion

The aim of the present study was to analyze and identify compensatory behavior in the context of German adolescents aged 11–15 years. Overall, in our study 55.5% of all opportunities were compensated (57 negatively and 52 positively). Of all compensatory behavior, negative compensatory behavior was observed slightly more often than positive compensatory behavior. Compensatory behavior in either direction (i.e., positive or negative) seemed to occur equally within-day and between-day, with no clear patterns emerging. The main enablers for compensating positively were friends/social support and influences for not compensating negatively were maintenance of the habitual schedule as well as hedonism/enjoyment of activities.

### Occurrence of compensation

Compensation occurred in just over half of possible compensation opportunities, with just over half of these occurrences being negative compensatory behavior. Whilst the occurrence of negative compensation was slightly more dominant than positive compensation in the present study, this trend was also observed in another study focusing on pre-adolescent girls [43]. In this study, all possible negative compensation opportunities were compensated negatively, whereas just 14.2% of possible positive compensation opportunities were positively compensated [43].

### Compensation timeframe and ratio

Overall, compensatory response can occur within-days, between-days as well as over (several) weeks [21–23], nevertheless, within our study we could only focus on compensation on a within-/between-day basis for one week. Regarding the timeframe of compensation in our study, data indicated that of all compensatory opportunities, 63.3% of all compensatory opportunities took place within-day. This was also stated in the study of Swelam et al. [31], who postulated that compensation scarcely occurred between-days because there is more like a reset each day. Nevertheless, this study did not include “real” data and instead participants were just asked in the interview about potential compensatory behavior. In general, this trend is in contrast with other findings [21, 22] stating that compensation mainly occurs between-days. However, Gomersall et al. [21] summarized more studies assessing between-day activity behavior compared to the review of Beck et al. [22], which could be a possible explanation for the discrepancy in the findings.

Regarding the ratio of compensation, the results across subjects were largely heterogenous, with high proportion of overcompensation (e.g., > 100% compensation), especially for positive compensation. One influencing factor for the heterogenous results could be enjoyment of PA as this seems to influence the degree of compensation [31]. For example, children positively compensate an increase of PA levels by 80% when they enjoyed the additional PA versus only a small partial compensation of < 50% when they did not enjoy the additional PA [35].

### Influencing factors for compensatory behavior

Overall, from the qualitative interviews we identified various factors from the qualitative interviews trying to explain activity compensation in adolescents. The most frequently mentioned influencing factor for negative compensation was related to relaxing after prolonged PA, either with or without being exhausted. In the first case, adolescents believed they could relax because they did more PA than usual before, even if they were not exhausted or tired. This could be explained by the missing awareness of their overall PA [44] in combination with the unawareness of health benefits of sufficient activity levels [18, 45]. In our study, health and training effects of the additional activity were also mentioned as one influence for not compensating the additional PA. Nevertheless, only few adolescents did not compensate additional PA by less PA at another time point. Thus, improving awareness of activity and health benefits may therefore be a crucial initial component of promotion campaigns, even if there are a few interventions that already consider this point [18, 46–48]. In the second case, there were also situations in which adolescents reported tiredness, sore muscle, or overexertion. These symptoms occurred mostly after bigger events like one-day tournaments in a specific sport (external influence). This influencing factor was also identified in the Australian study with elementary school children [31] and is also in line with the underlying mechanism of the ActivityStat hypothesis that each individual has a level of PA that is tolerable and PA above that tolerance threshold would be compensated [21]. In this respect, there may be instances where, what we've considered as 'negative compensation', serves as an important and biologically necessary mechanism to manage fatigue. The consideration of such nuances may be warranted in future intervention design and compensation research.

Regarding negative compensation, an interesting point was that negative compensation did not occur when the following activities were obligatory (e.g., physical education, training) or as a result of parental coercion. In this context of compensatory responses due to obligation or force, we would like to mention that the ActivityStat hypothesis does not totally fit in. The hypothesis is not suitable to explain thise cases in which compensation does not occur due to a biological mechanism. Here, compensation rather occurs due to external force. However, compensation due to self-obligating activities which are accompanied by enjoyment of the activity, is in line with Rowlands et al. [49] who argued that “hedonistic activities” (e.g. such as playing in a sports team) may override the biological control. Further, adolescents also mention that they want to stick to their routines and thus, did not cancel any of their activities to compensate the past additional activities. This finding is consistent with a previous study that reported that routines (i.e., overall and organized activities) helped children maintain a level of after school activity, regardless of whether they had been more active than usual at school [31]. Lastly, the results suggested that adolescents see each day as a new day, and thus, they do not adjust their PA the next day. Similar findings were reported by Swelam et al. [31], which explained this phenomenon as a ‘reset’ at the end of the day. In summary, it seems that routines as well as (self-)obligatory activities may be important indicators in avoiding negative compensation.

Besides the negative compensation, compensation occurred also in a positive direction. In this context, external influences were mentioned more often than internal influences. As external factors, social support was the most mentioned influence for positive compensation. In contrast, in cases where friends didn’t have time for co-participation, positive compensation was less likely. This is in line with existing literature stating that social support of peers is an important determinant and facilitator of PA [50–53]. Similar, Swelam et al. [31] indicated the relevance of family and friends also as influences for compensating positively. Thus, it seems that social support is also a facilitator of positive compensation. Lastly, good weather was also a factor that fostered positive compensation. Good weather is one of the strongest predictors of PA in children and adolescents [54–56]. Bad weather was identified to have the opposite effect [31], and may hinder children and adolescents being physically active and thus, compensating positively.

In the context of internal influences for positive compensations few adolescents mentioned a need for activity after a decrease of PA levels. However, these adolescents could not trace back where it was stemming from. Similar mechanisms were found in Australian elementary school children whose parents reported that their children had a ‘need’ to do more PA in response of low PA levels [31]. In that study, this was expressed by the parents as “just how [their child] was feeling”. In the current study, adolescents mentioned a kind of feeling and desire for doing activities which was supported the findings of the previous study. Overall, this behavior typically occurred spontaneously and may also be related to the (lack of) perception of compensation. This finding would also be supportive and indicative of the hypothesized biological control of PA, as it was hypothesized that, "A biologic regulator of physical activity in humans could operate by controlling the level of spontaneous, mainly involuntary PA (NEAT) or by increasing motivation to participate in planned voluntary activities." [57] (p.122).

In summary, the findings of this investigation assume kind of a malleability of the ActivityStat hypothesis with the ActivityStat hypothesis postulating not a rigid mechanism that is amenable to external influencing factors, such as environmental or social influences. We can consider this as a complex interaction of needs or desires for movement or rest with one’s (social and physical) environment (e.g., social support, weather, routines). For further exploration of compensation thresholds and timeframes a combination of objective and subjective measurement methods is needed to get deeper insights on these mechanisms.

### Awareness of compensation

In the present study, only few adolescents were aware that they compensated their increased/decreased behavior. It seems that it is more a situational behavior which is not consciously performed because of the awareness of less/more activity than usual before. This is in contrast with a qualitative study from Australia [31]. In this study, most elementary school children perceived their compensatory behavior while only some participants lacked awareness of any compensatory response or thought that compensatory behavior did not take place. The differences between these two studies could be due to differences in the study design. In the present study, data was driven from weekly schedules as well as diaries whereas in the study of Swelam et al. [31] the participants were asked to think about a hypothetical day they would have had lots of activity at school and how this would influence their activity behavior after school (same for between-day). As this is not based on “real” data, this is limited to the potential difference between claim and reality.

### Practical implications

The findings of the present study may also have some important practical implications. Firstly, social support seems to be helpful in avoiding negative compensation following an increase of PA, as well as supportive of positive compensation following less PA than usual. Further, it is important that adolescents find activities they like to do as such “hedonistic activities” seem to override the biological control. Thus, these activities may subsequently result in less negative compensatory behavior, especially in the instance of habitual hedonistic activities. In this context, routines may also be important in avoiding negative compensation as adolescents want to stick to their routines. As such, further support for adolescents in finding active routines may be warranted.

### Strengths & Limitations

The study’s novelty is grounded in examining actual compensatory behavior and subsequent influencing factors of compensatory behavior in adolescents. Despite the small sample size (*N* = 15), a high volume of activity data was yielded (382 activities overall). That makes it possible to differentiate between positive, negative and no compensation within the sample and within subjects. In addition, the used diary was programmed exactly for our research question and enables us to achieve the aim. This is also the first study, assessing the amount of deviation as well as the amount of the compensatory behavior.

However, there are several limitations that should be stated. Overall, the sample size (*N* = 15) is quite small. Given the data collection methodology (i.e., smartphone-based app), adolescents without a smartphone could not participate in the study. Further, the habitual weekly schedule was self-reported and occurred as a once-off assessment (i.e., in one week rather than across several weeks). As such, this could be associated with social desirability by indicating more PA behaviors than usual. Furthermore, this method did not allow for checking the validity of the habitual behavior. In this context, the subjective and retrospective assessment of activities within the weekly schedule and the diary carries the risk for recall bias. Compensatory analysis in our study could only be conducted on a within-/between-day basis for one week, and not on timeframes greater than one week, even if this is also a potential timeframe for compensation [21]. Additionally, we did not consider sedentary behaviors, even if this is important as compensation could also occur with regards to sedentary behaviors. Lastly, the Youth Compendium of PA [34] was utilized to calculate the MET cost for each activity. Nevertheless, if an activity wasn’t listed in the Youth Compendium, the MET cost was estimated based on similar activities, that could have limited the accuracy of the data analysis,

## Conclusion

This study provided new insights into occurrence and influencing factors for compensatory behavior in adolescents. Overall, 55.1% of deviations were compensated. The occurrence of positive and negative compensatory behavior were similar, with no obvious patterns emerging. Compensation occurred within-day slightly more often than between-day, which may be related to the mental presence of the activity as well as the reset at the end of the day. Overall, it seems that compensation is a complex interaction between biological control and influencing factors. For instance, social support seems to facilitate positive compensation, whilst routines and hedonistic activities may assist in avoiding negative compensatory behavior. Thus, it seems to be helpful to support individuals in their search for hedonistic activities as well as in the establishment of routines.

### Supplementary Information

**Additional file 1. **Weekly Schedule.

**Additional file 2. **Interview Guide – Compensation Qualitative Study.

**Additional file 3. **Calculation of Compensation.

**Additional file 4. **Amount (range) of deviation and compensation for positive and negative compensation for overall, boys and girls as well as within- and between-day.

**Additional file 5. **Prevalence (%) of low, medium and high partial positive compensation as well as positive overcompensation.

**Additional file 6. **Prevalence (N) of positive and negative compensation within- and between-day.

**Additional file 7. **Prevalence (%) of low, medium and high partial negative compensation as well as negative overcompensation.

**Additional file 8. **Amount (range) of deviation that was not compensated.

## Data Availability

The datasets used and/or analysed during the current study are available from the corresponding author on reasonable request.
