# Supplementary material for: Compensatory behavior of physical activity in adolescents – a qualitative analysis of the underlying mechanisms and influencing factors
Source: BMC Public Health. 2024 Jan 11;24:158. doi: 10.1186/s12889-023-17519-1 (PMC10785364; doi:10.1186/s12889-023-17519-1)
Supplement: Supplementary file 1 — Additional file 1. Weekly Schedule. [file 12889_2023_17519_MOESM1_ESM.pdf]

Additional file 1: Weekly Schedule

Dear study participants,

Thank you for your interest in the study. As you have already learned from the information letter, we aim to gather information about your physical activity habits. In this initial phase, it is essential for us to obtain a detailed overview of your weekly routine. It is important that you share all activities, whether they involve sitting, moving, exercising, attending school, or any other relevant activities. This will enable us to construct an accurate representation of your typical week.

Below, you will find two examples of schedules that illustrate how you can provide this information. Please start with your waking time and conclude with your bedtime There should be no time gaps, meaning each activity should be followed by the next one chronologically. If the provided lines are not sufficient, please feel free to add more. If you have any questions, please don't hesitate to contact Franzi Beck at franzi.beck@fau.de.

Thank you for your participation.

Activity Descriptions:

Below is a summary of the 5 intensity levels along with illustrative activity examples:

**Sleeping**	**inactivity**	**light intensity**	**medium intensity**	**Maximum intensity**
☐ Sleeping periods<br>☐ Without smart-phone/tablet	☐ Periods without phy-sical activity<br>☐ Watching TV/playing games<br>☐ Being on the phone<br>☐ Sitting in school/while doing homework	☐ Going for a walk<br>☐ Bike riding (slow)<br>☐ Activity without sweating or breath-lessness	☐ Any type of sport<br>☐ Practice, jogging, playing soccer<br>☐ Playing tag<br>☐ Sweating or being out of breath a little	☐ Work out<br>☐ Complete exhaustion<br>☐ Sweating a lot or be-ing out of breath heavily

Example of a weekly schedule:

Monday

**time**	**activity**	**intensity**				
Be as specific as possible about the time/duration of the activites	*Describe your activity as accurately as possible in a few words*	*Sleeping*	*Inac-tive/a-wake*	*light (e.g,. go-ing for a stroll)*	*Medium (e.g.doing-Sports)*	*Maximum*
6:15-6:45 am	getting up and ready for work			x		
6:45-7:00 am	riding the bike to work (1,5km)			x		
7:00-12:00 am	work: mainly sitting down		x			
12:00-12:20 pm	Going for a stroll with colleagues			x		
12:20-12:45 pm	Lunch		x			
12:45-4:30 pm	work: mainly sitting down		x			
4:30-4:40 pm	riding the bike back home (1,5km)			x		
5:00-5:15 pm	Driving car to Spardorf (swimming pool)		x			
5:20-6:15 pm	Going for a swim (2km)				x	
6:30-6:45 pm	Driving car back home		x			
7:00-8:00 pm	Cooking and eating			x		
8:00-10:30 pm	relaxing		x			
from 10:30 pm	sleeping	x				

Tuesday

time	activity	intensity				
Be as specific as possible about the time/duration of the activites	*Describe your activity as accurately as possible in a few words*	*Sleeping*	*Inac-tive/a-wake*	*light (e.g,. go-ing for a stroll)*	*Medium (e.g.doing-Sports)*	*Maximum*
6:15-6:45 am	getting up and ready for work			X		
6:45-7:00 am	riding the bike to work (1,5km)			X		
7:00-12:00 am	work: mainly sitting down		X			
12:00-12:20 pm	teaching – mainly standing activity		X			
12:20-12:45 pm	Going for a stroll with colleagues			X		
12:45-4:30 pm	Lunch		X			
4:30-4:40 pm	work: mainly sitting down		X			
5:00-5:15 pm	riding the bike back home (1,5km)			X		
5:20-6:15 pm	Riding bike to gym (5km)				X	
6:30-6:45 pm	Volleyball practice				X	
7:00-8:00 pm	Riding bike back home (5km)				X	
8:00-10:30 pm	Taking shower, cooking and eating, relaxing		x			
from 10:30 pm	Sleeping	x				

Weekly schedule from ____(register ID)

Monday

time	activity	intensity				
Be as specific as possible about the time/duration of the activites	*Describe your activity as accurately as possible in a few words*	*Sleeping*	*Inac-tive/a-wake*	*light (e.g,. go-ing for a stroll)*	*Medium (e.g.doing-Sports)*	*Maximum*

Tuesday

time	activity	intensity				
Be as specific as possible about the time/duration of the activites	*Describe your activity as accurately as possible in a few words*	*Sleeping*	*Inac-tive/a-wake*	*light (e.g,. go-ing for a stroll)*	*Medium (e.g.doing-Sports)*	*Maximum*

Mittwoch

time	activity	intensity				
Be as specific as possible about the time/duration of the activites	*Describe your activity as accurately as possible in a few words*	*Sleeping*	*Inac-tive/a-wake*	*light (e.g,. go-ing for a stroll)*	*Medium (e.g.doing-Sports)*	*Maximum*

Thursday

**time**	**activity**	**intensity**				
Be as specific as possible about the time/duration of the activites	*Describe your activity as accurately as possible in a few words*	*Sleeping*	*Inac-tive/a-wake*	*light (e.g,. go-ing for a stroll)*	*Medium (e.g.doing-Sports)*	*Maximum*

Friday

time	activity	intensity				
Be as specific as possible about the time/duration of the activites	*Describe your activity as accurately as possible in a few words*	*Sleeping*	*Inactive/awake*	*light (e.g,. going for a stroll)*	*Medium (e.g.doing-Sports)*	*Maximum*

Saturday

time	activity	intensity				
Be as specific as possible about the time/duration of the activites	*Describe your activity as accurately as possible in a few words*	*Sleeping*	*Inac-tive/a-wake*	*light (e.g,. go-ing for a stroll)*	*Medium (e.g.doing-Sports)*	*Maximum*

Sunday

time	activity	intensity				
Be as specific as possible about the time/duration of the activites	*Describe your activity as accurately as possible in a few words*	*Sleeping*	*Inac-tive/a-wake*	*light (e.g,. go-ing for a stroll)*	*Medium (e.g.doing-Sports)*	*Maximum*
