# Supplementary material for: Compensatory behavior of physical activity in adolescents – a qualitative analysis of the underlying mechanisms and influencing factors
Source: BMC Public Health. 2024 Jan 11;24:158. doi: 10.1186/s12889-023-17519-1 (PMC10785364; doi:10.1186/s12889-023-17519-1)
Supplement: Supplementary file 2 — Additional file 2. Interview Guide – Compensation Qualitative Study. [file 12889_2023_17519_MOESM2_ESM.pdf]

Additional File 2: Interview Guide – Compensation Qualitative Study

Interviews are based on the comparison between the typical weekly schedule and the movement diary over the course of a week. Three scenarios may arise:

1. Positive Compensation

Adolescents have shown a decrease in physical activity in the movement diary compared to the weekly schedule.

However, this decrease was compensated by an increase in physical activity during another time period.

Interview guide questions:

- You moved less than usual on XY day (referring to the specific situation in the movement diary).
- Why was it less than usual?
- How was it for you? How did you feel before and after?
- You then moved more than usual the next day (referring to the movement diary):
- How did you feel during that time? How did you feel before and after?
- Why did you decide to move more after a reduction (referring to the situation)?

2. Negative Compensation

Adolescents have shown an increase in physical activity in the movement diary compared to the weekly schedule.

However, this increase was compensated by a decrease in physical activity during another time period.

Interview guide questions:

- You moved more than usual on XY day (referring to the specific situation in the movement diary).
- Why was it more than usual?
- How was it for you? How did you feel before and after?
- You then moved less than usual the next day (referring to the movement diary):
- How did you feel during that time? How did you feel before and after?
- Why did you decide to move less after an increase (referring to the situation)?

3. No Compensation

Adolescents have not compensated for an increase or decrease in physical activity.

They remained consistently active/inactive over the duration.

Interview guide questions:

- You increased/reduced your activity at a certain time (referring to the movement diary), but did not compensate by moving less/more afterward.
- Why didn't you balance that out?
- Who decided that you engage in this physical activity?
- How did you feel during each movement?
