# Supplementary figures and images for: Compensatory behavior of physical activity in adolescents – a qualitative analysis of the underlying mechanisms and influencing factors
Source: BMC Public Health. 2024 Jan 11;24:158. doi: 10.1186/s12889-023-17519-1 (PMC10785364; doi:10.1186/s12889-023-17519-1)

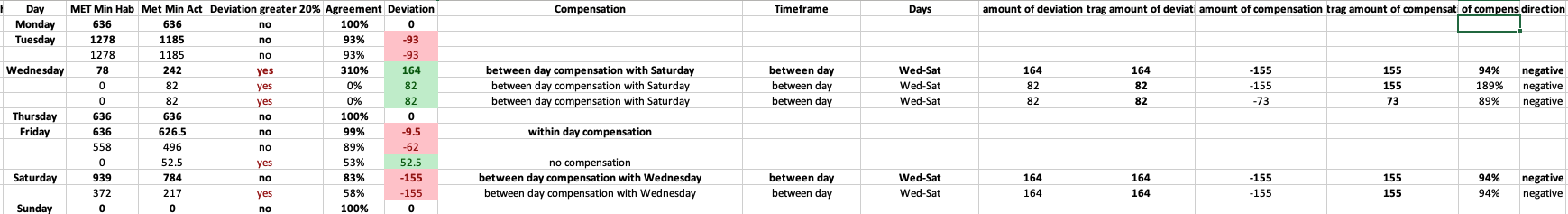

Supplement: Supplementary file 3 — Additional file 3. Calculation of Compensation. [file 12889_2023_17519_MOESM3_ESM.png]
