# Supplementary material for: Compensatory behavior of physical activity in adolescents – a qualitative analysis of the underlying mechanisms and influencing factors
Source: BMC Public Health. 2024 Jan 11;24:158. doi: 10.1186/s12889-023-17519-1 (PMC10785364; doi:10.1186/s12889-023-17519-1)
Supplement: Supplementary file 4 — Additional file 4. Amount (range) of deviation and compensation for positive and negative compensation for overall, boys and girls as well as within- and between-day. [file 12889_2023_17519_MOESM4_ESM.pdf]

Additional File 4: Amount (range) of deviation and compensation for positive and negative compensation for overall, boys and girls as well as within- and between-day

	**Within-day**			**Between-day**			**overall**		
	**Positive**	**negative**	**overall**	**Positive**	**negative**	**overall**	**Positive**	**negative**	**overall**
overall									
**MET-minutes of activity that is compensated Mean (SD) (Range)**	-227.88 (257.12) (-31 to -963.5)	746.56 (802.85) (49.5 to 2433)	490.98 (650.07) (31 to 2433)	-274.6 (433.4) (-54 to -1896)	397.59 (397.59) (16.5 - 16.5)	342.2 (390.5) (16.5 to 1896)	-244.1 (325.4) (-31 to -1896)	611.87 (683.54) (16.5 to 2433)	436.4 (571.3) (16 to 2433)
**MET-minutes of compensating activity Mean (SD) (Range)**	314.06 (264.03) (24.5 to 750)	-542.74 (400.61) (-48 to -1164)	430.06 (356.84) (24.5 to 1164)	324.1 (397.7) (48.5 to 1210)	307.45 (307.45) (35 to 35)	314.9 (375.5) (35 to 1752)	317.5 (312.8) (24.5 to 1210)	-451.93 (401.14) (-35 to -1752)	387.8 (366.3) (24.5 to 1752)
**Amount of compensation**	137.8%	72.9%	87.6%	130.4%	77.3%	90.1%	129.2%	73.8%	88.3%
boys									
**MET-minutes of activity that is compensated Mean (SD) (range)**	-229.27 (267.94) (-31 to -963.5)	780.08 (835.02) (94.5 to 2433)	474.07 (646.68) (31 - 2433)	-232.5 (177.0) (-54 to -525)	416.94 (381.63) (24 to 1269)	320.3 (300.2) (24 - 1269)	-230.1 (244.7) (-31 to -963.5)	673.28 (744.24) (24 to 2433)	431.0 (573.3) (24 to 2433)
**MET-minutes of compensating activity Mean (SD) (Range)**	316.4 (264.28) (24.5 to 750)	-635.83 (416.99) (-53 to -1164)	458.37 (373.29) (24.5 to 1164)	170.5 (142.3) (58 to 504)	-453.38 (498.19) (-36 to -1752)	305.2 (377.9) (36 to 1752)	277.3 (244.9) (24.5 to 750)	-582.17 (442.72) (-36 to -1752)	415.5 (378.4) (24.5 to 1752)
**Amount of compensation**	138.0%	81.5%	96.7%	58.5%	108.7%	91.5%	118.9%	86.5%	95.7%
Girls									
**MET-minutes of activity that is compensated Mean (SD) (Range)**	-217.5 (181.87) (-60 to -375)	673.41 (761.08) (49.5 to 1860)	551.83 (681.46) (49.5 to 1860)	-340.79 (686.69) (-58.5 to - 1896)	381.46 (342.07) (16.5 to 1034.5)	366.47 (478.64) (16.5 to 1896)	-295.95 (544.72) (-58.5 to - 1896)	521.09 (586.54) (16.5 to 1896)	448.25 (575.06) (16.5 to 1896)
**MET-minutes of compensating activity Mean (SD) (Range)**	296.5 (301.95) (35 - 558)	-339.64 (282.09) (-48 to -774)	328.13 (277.07) (35 to 774)	565.57 (550.56) (48.5 to 1210)	-185.83 (128.82) (-35 to -358.5)	325.74 (382.89) (35 to 1210)	467.73 (477.13) (35 to 1210)	-259.39 (225.03) (-35 to -774)	326.79 (335.46) (35 to 1210)
**Amount of compensation**	136.3%	50.4%	59.5%	166.0%	48.7%	88.9%	158.0%	49.8%	72.9%
