# Supplementary material for: Compensatory behavior of physical activity in adolescents – a qualitative analysis of the underlying mechanisms and influencing factors
Source: BMC Public Health. 2024 Jan 11;24:158. doi: 10.1186/s12889-023-17519-1 (PMC10785364; doi:10.1186/s12889-023-17519-1)
Supplement: Supplementary file 5 — Additional file 5. Prevalence (%) of low, medium and high partial positive compensation as well as positive overcompensation. [file 12889_2023_17519_MOESM5_ESM.pdf]

Additional file 5: Prevalence (%) of low, medium and high partial positive compensation as well as positive overcompensation

	**Low compensation**	**Medium compensation**	**High compensation**	**Overcompensation**
**Overall**				
Overall	21.1	7.7	7.7	63.5
Within	20.6	8.8	0	73.5
Between	22.2	11.1	22.2	44.4
**Boys**				
overall	24.4	2.4	7.3	65.9
Within	23.3	0	0	76.7
Between	27.3	9.1	27.3	36.3
**Girls**				
Overall	9.1	27.3	9.1	54.5
Within	0	50.0	0	50.0
Between	14.3	14.3	14.3	57.1
