# Supplementary material for: Compensatory behavior of physical activity in adolescents – a qualitative analysis of the underlying mechanisms and influencing factors
Source: BMC Public Health. 2024 Jan 11;24:158. doi: 10.1186/s12889-023-17519-1 (PMC10785364; doi:10.1186/s12889-023-17519-1)
Supplement: Supplementary file 6 — Additional file 6. Prevalence (N) of positive and negative compensation within- and between-day. [file 12889_2023_17519_MOESM6_ESM.pdf]

Additional file 6: Prevalence (N) of positive and negative compensation within- and between-day

	**Within-day**			**Between-day**			**overall**
	**overall**	**positive**	**negative**	**overall**	**positive**	**negative**	
**Boys**	54	30	24	21	11	10	75
**Girls**	15	4	11	19	7	12	34
**overall**	69	34	35	40	18	22	109
