# Supplementary material for: Compensatory behavior of physical activity in adolescents – a qualitative analysis of the underlying mechanisms and influencing factors
Source: BMC Public Health. 2024 Jan 11;24:158. doi: 10.1186/s12889-023-17519-1 (PMC10785364; doi:10.1186/s12889-023-17519-1)
Supplement: Supplementary file 7 — Additional file 7. Prevalence (%) of low, medium and high partial negative compensation as well as negative overcompensation. [file 12889_2023_17519_MOESM7_ESM.pdf]

Additional file 7: Prevalence (%) of low, medium and high partial negative compensation as well as negative overcompensation

	**Low compensation**	**Medium compensation**	**High compensation**	**Overcompensation**
**Overall**				
Overall	33.3	7.0	17.6	42.1
Within	34.3	5.7	17.1	42.9
Between	31.8	9.1	18.2	40.9
**Boys**				
overall	29.4	2.9	14.7	53.0
Within	33.3	0	5.9	50.0
Between	20.0	10.0	30.0	60.0
**Girls**				
Overall	56.5	4.3	34.8	4.4
Within	54.5	0	45.5	0
Between	58.4	8.3	25.0	8.3
