# Supplementary material for: Compensatory behavior of physical activity in adolescents – a qualitative analysis of the underlying mechanisms and influencing factors
Source: BMC Public Health. 2024 Jan 11;24:158. doi: 10.1186/s12889-023-17519-1 (PMC10785364; doi:10.1186/s12889-023-17519-1)
Supplement: Supplementary file 8 — Additional file 8. Amount (range) of deviation that was not compensated. [file 12889_2023_17519_MOESM8_ESM.pdf]

Additional file 8: Amount (range) of deviation that was not compensated

	overall	boys	Girls
**Negative deviation**			
Range	-26.0 to -1488.0	-26 to -1488	-35.0 to -945.0
Mean (SD)	-267.5 (292.3)	-359.3 (366.5)	-200.1 (201.6)
**Positive deviation**			
Range	52.5 to 480	88.5 to 480.0	52.5
Mean (SD)	194.0 (195.3)	241.2 (209.5)	
